# The Effect of a Mixed Circuit of Aerobic and Resistance Training on Body Composition in Older Adults—Retrospective Study

**DOI:** 10.3390/ijerph18115608

**Published:** 2021-05-24

**Authors:** Anna Pieczyńska, Ewa Zasadzka, Tomasz Trzmiel, Małgorzata Pyda, Mariola Pawlaczyk

**Affiliations:** 1 Department of Occupational Therapy, Poznan University of Medical Sciences, 60-781 Poznań, Poland; apieczynska@ump.edu.pl (A.P.); ttrzmiel@ump.edu.pl (T.T.); 2 First Department of Cardiology, Poznan University of Medical Sciences, 61-848 Poznań, Poland; malgorzatapyda@ump.edu.pl; 3 Department and Division of Practical Cosmetology and Skin Diseases Prophylaxis, Poznan University of Medical Sciences, 60-623 Poznań, Poland; mariolapawlaczyk@ump.edu.pl

**Keywords:** body composition, exercise training, older adults, physical activity

## Abstract

Ageing is inevitably associated with body composition changes, such as loss of muscle mass, increase in the total fat mass, and unfavorable reduction of subcutaneous fat. Physical activity exerts significant effects on the body composition. The aim of the study was to investigate the effects of two different weekly doses of resistance-aerobic training on the body composition in older people. The study consisted in a retrospective data analysis of fitness club members aged ≥60. The trainees participated in resistance-aerobic training sessions two or three times/week for a minimum of two months. A body composition analysis was performed before and after the training sessions. Group 1 (36 subjects) and Group 2 (28 subjects) had two and three training sessions/week, respectively. A higher skeletal muscle mass was found in Group 1 and lower waist-hip-ratio indices were observed in Group 2. No statistically significant differences were found in the body mass, skeletal muscle mass, fat mass, total body water, lean mass, body mass index, visceral fat area between both groups. The number of training session/week proved to be statistically insignificant for all investigated variables. Resistance-aerobic training with two sessions/week may be as effective in maintaining proper body composition in older people as the same training at the dose of three sessions/week.

## 1. Introduction

Ageing is inevitably associated with unfavorable changes in body composition, such as loss of muscle mass, increase in the total fat mass, and reduction of subcutaneous fat. Accumulation of visceral fat, liver fat, and muscle fat infiltration also accompany the process of ageing [1]. Body composition has been demonstrated to affect numerous health aspects in older subjects, e.g., higher muscle mass lowers the risk of motor handicap [2]. Muscle fat infiltration has been linked to higher mortality [3], while excessive waist circumference may lead to frailty [4]. Ageing-related changes in body composition (muscle mass loss and accumulation of fat mass) may result in sarcopenic obesity, which is an increasingly common occurrence in ageing populations [5].

Thus, maintenance of proper body composition seems to be a particularly important aspect of healthy ageing. According to a systematic review and a meta-analysis of the available literature conducted by Liberman et al. [6], physical activity exerts a significant effect on body composition among older populations. In addition, these authors emphasize the need for further analysis of various methods of training.

Advanced age is associated with a higher risk for coronary heart diseases and the related mortality. Older people are more likely to experience complications after a cardiac incident or major surgery due to low fitness and activity [7]. Physical activity, whose beneficial effects on health in that age group have been demonstrated by numerous studies, may result in improved indices of cardiovascular health [8,9], but it is vital to select a suitable type of physical activity. 

Resistance training is among the most widely recommended forms of physical activity for older populations. It slows down the loss of muscle mass [10,11,12], and favorably affects functional independence [13], cognitive abilities [14] and self-esteem [15]. Resistance training may prevent the onset of type-2 diabetes or play a role in its treatment, lower blood pressure, regulate lipid balance, and increase bone tissue density [16]. The literature offers reports that resistance training may also alleviate ageing-related limitations in functional mobility, improve static and dynamic balance, as well as lower the risk of falling [10].

Aerobic (cardio) training, which increases aerobic capacity, lowers blood pressure [17,18] and serum lipid levels [19]—resulting in higher cardiovascular capacity [20]—is another important form of physical activity. In addition, it increases the insulin sensitivity by reducing the fat mass [21]. Combined training has also a beneficial effect on cardiometabolic risk factors, hemodynamics, and physical performance in the elderly group [22]. Both, endurance and resistance training display beneficial effects on arterial size, function, and wall thickness [23]. Therefore, it seems advisable to combine these types of training in older subjects [24]. Exercise lowers the risk of death due to cardiovascular causes by exerting a beneficial effect on the vascular system, such as a better endothelial function and a better compliance of the vessel (reduced stiffness) [25]. Regular physical exercise decreases the resting heart rate, blood pressure, atherogenic markers, and increases the physiological cardiac hypertrophy [26]. Several recent studies demonstrated that physical activity is associated with lowered markers of inflammation, improved metabolic health, less risk for cardiac failure, and higher chances of survival [27,28,29]. In addition, physical activity has a positive effect on the pulmonary function and cardiorespiratory fitness, both of which may decrease with age [30,31].

The best health effects are achieved if both, type and dose of physical activity are taken into consideration. According to the World Health Organization (WHO) [32], people aged ≥65 years should undertake at least 75 min/week of high-intensity aerobic exercise or 150 min/week of moderate-intensity aerobic exercise, or the equivalent of a combination of both. The WHO also recommends varied multicomponent physical activity that emphasizes functional balance and strength training at moderate or greater intensity, on three or more days a week, to enhance functional capacity and to prevent falls [32]. Regular, moderate physical activity of older people improves their physical performance and helps prevent the frailty syndrome [33]. An example of mixed training is our mixed circuit, which consists of eight strength exercises to strengthen the main muscle groups and two aerobic exercises (cross walker and ergometer). There are several reasons why it is prudent to attempt and define the optimal dose of training for older trainees, chief among them the fact that excessive training will seem as an unattainable goal, and as such will be discouraging, whereas an insufficient amount of training will not bring the desired health effects. Guidelines regarding the number of training sessions might encourage older people to undertake regular exercise, resulting in optimal health effects. 

The aim of the study was to analyze the effect of two different regimens of strength-endurance training (two or three sessions per week) and its relationship with the following body mass parameters: skeletal muscle mass, fat mass, total body water, lean body mass, percent body fat, waist-hip ratio, and visceral fat area in the age group of ≥60 years.

## 2. Materials and Methods

### 2.1. Data Eligibility

**Figure 1:**
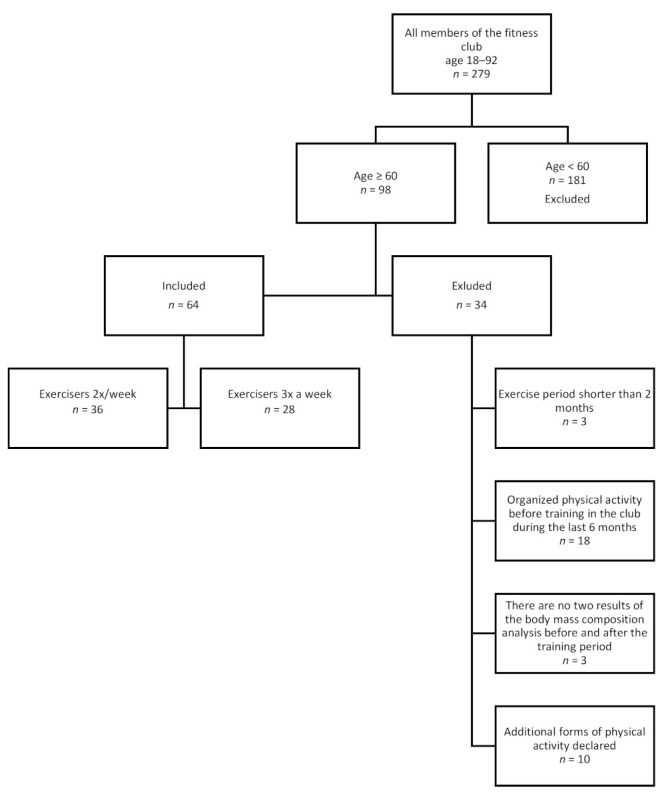
Criteria for including data for further analysis.

The study was retrospective in nature. We analyzed data from one of the fitness clubs in the city of Poznań (Poland, Wielkopolskie Region) about their trainees between 2017–2020. The study included city-dwellers (population: >500,000), with high social and economic status, secondary or higher education, no contraindications for strength-endurance training. Data collection and analysis for the purpose of the study was conducted in February 2020. The data had been collected by one trainer and anonymized before submission. Inclusion criteria: age ≥60 years, no health contraindications for training, ability to exercise properly and independently. Exclusion criteria: training lasting less than two months, lack of regularity in exercise, participation in additional sports activities, lack of body weight analysis before or after training, undertaking other structured exercises within 6 months before our study. The chart of data inclusion is presented in Figure 1. 

### 2.2. Description of the Resistance-Aerobic Training

One (circuit) training session lasted exactly 36 min for each study participant and included eight workout stations (2 for aerobic and 6 for resistance). The following workout stations were used:

- 1. Knee extensor muscles;
- 2. Chest muscle training;
- 3. Upper back muscle training;
- 4. Stationary bike;
- 5. Knee flexor muscles;
- 6. Abdominal muscle training;
- 7. Erector spinae training;
- 8. Elliptical trainer.

Resistance-aerobic exercises lasted 1 and 4 min, respectively, with a 30-s interval between the workout stations. Two circuits, with a recommended break of 1 min, were performed during a training session. The training group included a maximum of 8 participants at a given time.

**Figure 2:**
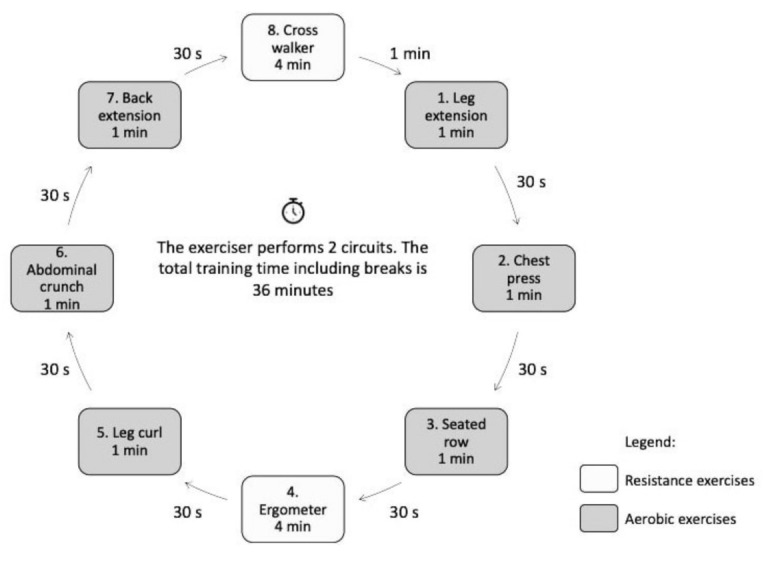
Training scheme.

The resistance exercise load was delivered by the machine during the concentric and eccentric muscle contraction. The tempo was set by coordinating the movements of the trainee with the graphic representation on the machine screen to ensure that each trainee performed 15 repetitions of a given exercise in the course of 1 min. The training plan is presented in Figure 2. 

The initial load value was individually adjusted during the first training session by measuring the maximal strength (it was possible due to electric engines which replaced standard weights) and was 40% of the mean maximum value. Each trainee started his or her training session with a higher eccentric contraction load, which was 130% of the concentric contraction value. As for aerobic training, the load was calibrated using a pulsometer for the exercises to be performed within the range of 60–70% of the maximum heart rate (HR max). The HR max was calculated using the following formula: HR max = 210 − (0.5 × age) − (0.022 × weight in kg) for women and HR max = 210 − (0.5 × age) − (0.022 × weight in kg) + 4 for men [34]. A certified physiotherapist-trainer supervised each training to monitor performance and load. Every two weeks, the load on the resistance machines was increased by 1 kg, if the condition of the trainee allowed it. 

### 2.3. Analysis of Body Composition

The body composition was evaluated with the bioelectric impedance analysis using the eight-point tactile electrode system InBody 170. The parameters directly measured by InBody are: impedance (Z), reactance (Xc), and phase angle. Z is the impedance, or resistance in the human body, Xc is the reactance which is the resistance in the condenser. The condenser in the human body is the cell membrane. Impedance is the sum of the vector of reactance (Xc) and resistance (R) and, when this sum is expressed in degrees, it is called a phase angle. The analyzer evaluates lean body mass and the percentage of fat mass on the basis of segmental multi-frequency analysis, separately for the torso and each limb. This equipment was used by other authors as well to determine the body composition of older subjects [35,36].

Description of the InBody machine and measurement performed:

- Direct segmental measurement of body mass composition;
- 8-point tetra-polar tactile electrode system (2 electrodes of the left foot, 2 electrodes of the right foot, 2 electrodes of the left hand, 2 electrodes of the right hand);
- Frequency—20, 100 kHz;
- Volume—200 uA;
- Measurement duration—30 s;
- Body mass limit—5–250 kg;
- Age limit—1–99;
- Height limit—50–250 cm.

The identification number, age, sex and height of the participants were entered into the analyzer. After all metal accessories and heavy clothing have been removed, the participants mounted the platform and positioned their feet on the designated spots with four electrodes, and griped the two handles (two electrodes in each handle, one for the thumb and one for the rest of the hand). The upper limbs were straight, abducted by 30–45 degrees from the torso. The machine provides information on the Body Mass Index (BMI), calculated as body weight quotient (in kg) to squared body height (in m2), skeletal muscle mass (SMM), fat mass (FAT), total body water (TBW), lean body mass (LBM), waist-hip ratio (WHR) and visceral fat area (VFA). The height (barefoot) and body weight (in light clothing) were measured with the accuracy of 0.1 cm and 0.1 kg, respectively. The measurements were taken by a certified dietitian. The subjects refrained from eating and drinking for a minimum of 8 h prior to the measurement. The analysis was always conducted in the morning. The participants were required to abstain from physical activity (no training sessions) one day before the measurements were taken.

### 2.4. Ethical Issues

Study approval and confirmation of its non-experimental nature were obtained from the Local Ethics Committee. The Declaration of Helsinki was followed. Written informed consent to use anonymized data of club members, who earlier consented to data collection by the club, was obtained from the club owner. 

### 2.5. Statistical Analysis 

Statistical analysis was conducted using the Jamovi software (v.0.8.1.5), (Jamovi project, Sydney, NSW, Australia). In the absence of normal distribution of the investigated variables, the Mann–Whitney test was used to compare the groups before training. The General Linear Model was used to control for the following variables: sex, age and number of months in training. The analysis was conducted to assess the effect of the number of training sessions per week on the body mass composition for each variable. The *p*-value of <0.05 was considered as statistically significant. A design with group sample sizes of 30 and 19, respectively, can detect effect sizes of δ ≥ 0.99 with a probability of at least 0.911, assuming a two-sided criterion for detection that allows for a maximum type I error rate of α = 0.05.

## 3. Results

A total of 64 subjects (36—Group 1 and 28—Group 2) were included in the study. Groups 1 and 2 had two and three training sessions/week, respectively. The majority of the participants were female (64% and 57% in groups 1 and 2, respectively). The time of participation in the training is presented in Table 1. This variable was controlled when testing the effect of the training frequency. The study population characteristics at baseline are presented in Table 2. No significant differences in age and body composition were found between both groups at baseline. The results at baseline and after the last training were compared between both groups (Table 3). The mean body weight, tissue mass and BMI were lower in all subjects. A reduced area of visceral fat and percentage of fat mass were observed. The mean water weight and lean body mass were slightly lower. A higher skeletal muscle mass was found in Group 1 (2 training sessions/week) and lower WHR indices were observed in Group 2 (3 training sessions/week). No statistically significant differences were found in the body mass, SMM, FAT, TBW, LBM, BMI, VFA (at baseline and after the last training) between both groups (Table 3). A statistically significant reduction in the percentage of body fat and WHR was observed in Group 2.

In addition, the effects of training frequency on body composition (controlled for sex, age and the number of months in training) were analyzed. The number of trainings/week proved to be statistically insignificant for all investigated variables (Table 4).

## 4. Discussion

The literature demonstrates a marked lack of consensus on the optimal frequency of trainings per week for older populations. In particular, the number of studies investigating the optimal dose of resistance-aerobic training, whose positive effects on health in older people have been reported by numerous authors, is limited. In our study, we confirmed the beneficial effects of resistance-aerobic training. We used the BIA method to analyze the body weight composition, which is a fast, safe and non-invasive method to obtain quantitative estimates of the body composition. A multifrequency BIA and, specifically, bioimpedance spectroscopy is preferred for fluid volume measurements, although for a general body composition assessment, the BIA at 50 kHz is more widely used [37]. In Group 1 (two sessions/week), the muscle mass increased on average by 5.9% (whereas in Group 2 no increase was observed). The body fat was reduced by 3.7% and 8.3% in Groups 1 and 2, respectively. A lower body fat percentage and reduction of the visceral fat area were also observed in both groups. Those changes may prove to be highly beneficial for older people, whose body composition has a crucial effect on the cardiovascular risk factors [5], and may prevent sarcopenic obesity [5,38,39].

Combination (resistance-aerobic) training had a favorable outcome in the entire study population, which is consistent with the reports of Timmons et al. [40] who demonstrated that resistance-aerobic training more effectively reduced the fat mass in older subjects (−2.6%) as compared to resistance training or aerobic training alone (−0.4% and −2.2%, respectively). These authors also proved its usefulness in increasing the gait speed and lower limb strength as compared to the other two types of training. Their study participants trained for 12 weeks, 3 times/week, for a total of 72 min. In the study by Lima et al. [41], who compared the impact of the resistance-aerobic training with only resistance or only aerobic training on body composition, the subjects also trained 3 times/week. A significantly reduced body mass, abdominal and waist circumference, and 24-h ambulatory monitored blood pressure were revealed in both, aerobic and resistance-aerobic training groups. However, the body fat was reduced only in the latter. Schroeder et al. [42] compared the effect of resistance, aerobic and combination training on the risk factors for cardiovascular diseases (CVD), including muscle strength and body composition in 69 adults (mean age: 58 ± 7 years) with elevated risk for CVD. Combination training (3 times/week for 60 min) resulted in a higher strength and lean body mass. Their study suggests that combination training may be more valuable than resistance training or aerobic training alone because it seems to have the most beneficial effect on the risk factors for CVD. The resistance training itself, performed 3 times a week may also reduce the risk of CVD in elder women as shown by Dos Santos et al. [43]. In their study, the participants were divided in two groups with different repetition zones. The results obtained for both groups indicated a lesser risk of developing a CVD in comparison with the control group not undergoing any training. 

To the best of our knowledge, our study has been the first to investigate two different frequencies of resistance-aerobic training on various body mass elements in a population of healthy older community-dwellers. No statistically significant changes in body composition as compared to the weekly number of training sessions (2 or 3 times/week) were found for any of the investigated variables, controlled for sex, age, and number of training weeks. Statistically significant changes between Groups 1 and 2 (2 and 3 sessions/week, respectively) were observed only for the waist-hip ratio/WHR (*p* = 0.006), which decreased only in Group 2 by 1.6%. The following parameters decreased in both groups: body weight, fat mass, visceral fat, percentage of fat tissue and BMI. Changes in muscle mass at baseline and after the last training were not statistically significant in both groups, although a 5.9% increase was detected in Group 1. 

Fisher et al. [44] investigated the frequency of resistance-aerobic training in older women in the following subgroups: 1 aerobic and 1 resistance training session/week (group 1), 2 aerobic and 2 resistance training sessions/week (group 2), and 3 aerobic and 3 resistance training sessions/week (group 3). Similar changes in the body mass composition (higher lean body mass and lower percentage of fat mass and BMI) were found in all three subgroups.

Most reports on the recommended training dose for older people concern resistance training. Nascimento et al. [45] investigated 45 women (age ≥60 years) who participated in resistance training for 12 weeks (8 exercises, 10–15 repetitions) and also found no differences between groups with 2 and 3 sessions/week. The muscle mass increase was comparable in both groups (+5.5% for 2 sessions/week and 5.8% for 3 sessions/week), but, unlike in our study, the change was statistically significant (*p* < 0.001). Similar changes in the lean body mass were reported by Murlasits et al. [46] for groups with different training frequency. They investigated 29 people over the age of 60 years undergoing resistance training 2 or 3 times per week, for 8 weeks. Pina et. al. [47] also found no change in the lean body mass in groups training 2 or 3 times per week. Ihalainen et al. [48] analyzed people aged 65–75 years who were assigned to groups training 1, 2 or 3 times/week and non-training controls, and found that a training frequency over 2 times/week may help maintain adequate fat mass levels. Turpela et al. [49] analyzed the effects of various frequencies of resistance training on body composition and functional capabilities in elderly subjects. A total of 106 people, aged 64–75 years, were randomly assigned to one of four groups (1, 2 or 3 times/week and non-training controls). All subjects from the three study groups participated in a full-body resistance training (2–5 series, 4–12 repetitions/exercise, 7–9 exercises/session) and no significant changes in the body composition were observed. These authors concluded that functional capabilities may be improved using the 2 sessions/week regime. The advantage of resistance training 3 times a week over training once a week in an obese female population was demonstrated by Toselli et al. [50]. The authors showed that, although body weight, waist circumference and body fat percentage decreased statistically significantly in both groups, only in the group training 3 times a week there was also an increase in muscle strength (measured with a hand dynamometer) and the phase angle. Huang et al. [51] demonstrated that medium-intensity aerobic training (66–73% heart rate reserve, 40–50 min/session, 3–4 days/week) is an optimal and effective dose for maximal cardiorespiratory benefits in healthy sedentary older adults. In our study, no statistically significant changes in body composition between Groups 1 and 2 were observed, although higher muscle mass was detected only in Group 1, which may be indicative of an advantage of the 2 over 3 sessions/week training regime. As mentioned above, the literature lacks consensus on the optimal training dose and conflicting reports, recommending 2 as well as 3 sessions/week, may be found. 

The search for the optimal training dose seems to be especially important as far as older populations are concerned. Time is one of the barriers to physical activity in that age group [52], so medium-frequency training, e.g., 2 sessions/week, might be an incentive for more older people to engage in physical activity, while still preserving all benefits of the regular training. Fisher et al. [53], in their review, recommend the minimal training dose (e.g., ≤60 min, 2 times/week), which may later be adjusted, if necessary. These authors demonstrated that even simple resistance training at the dose of <60 min, 2 times/week, is sufficient to be beneficial for the health. They also emphasize the necessity of breaks and regeneration between the sessions, so as not to cause overtraining, which will not be the case if minimal training doses (2 times/week) are recommended. The findings of our present study indicate that similar recommendations may be beneficial also in case of resistance-aerobic training. The training, apart from the abovementioned health benefits, is also associated with another positive aspect: the exercises are performed in groups of 8 people, allowing older subjects, who often feel lonely, to acquire and foster new social contacts. Other authors point to physical activity as a factor which either protects against the development of depressive symptoms and fear, or lowers their intensity among other people, consequently improving their quality of life [54,55,56]. Kekäläinen et al. [57] claim that participation in resistance training sessions 2 times/week is the most beneficial for various aspects of psychical functioning of older populations. 

Our study is not without limitations, chief among them is the lack of control over the dietary habits and daily physical activity, e.g., the number of steps/day of the study participants. Despite the fact that the subjects were instructed not to engage in additional exercises, their daily physical activity levels connected with normal everyday functioning, may have impacted the final results. In addition, most investigations which were used as a reference for our findings conducted body measurements with Dual-energy X-ray Absorptiometry (DXA), whereas our analysis was based on the bioelectric impedance method. The measurement tool used in our study calculates the bioimpedance. However, in recent years, the bioimpedance vector analysis (BIVA) has been increasingly used for the assessment of body composition. BIVA is considered to be free from errors associated with prediction equations as it interprets the raw bioimpedance value resistance (R) and reactance (Xc) [58].

## 5. Conclusions

This study demonstrated that resistance-aerobic training (2 sessions/week) may be as effective in maintaining proper body composition in older people as the same training at the dose of 3 sessions/week. It is a novel approach as other authors have focused on defining the optimal dose of resistance training alone. In addition, evidence that 2 rather than 3 trainings will suffice to bring about positive health changes may help and break the time-barrier argument and encourage more older subjects to undertake physical activities. Further large-sample studies to investigate the resistance-aerobic training, whose beneficial effects for the health of older populations were confirmed by numerous sources, are necessary.

## Figures and Tables

**Table 1:** Time of participation in training in months.

Group	Mean	SD	Median
<6 months	2.15	2.02	2
6–12 months	9.51	1.71	10
>12 months	20.58	7.40	18

Abbreviations: SD—standard deviation.

**Table 2:** Participant characteristics at baseline.

Parameters (m ± SD)	Training 2x/week			Training 3x/week			*p* ^a^
	Total *n* = 36	Women *n* = 23	Men *n* = 13	Total *n* = 28	Women *n* = 16	Men *n* = 12	
Age years	69 ± 6	68 ± 5	71 ± 8	71 ± 7	72 ± 8	70 ± 5	0.255
Body mass kg	81.0 ± 16.6	76.8 ± 14.5	88.6 ± 18.1	81.2 ± 12.3	77.6 ± 10.5	86.0 ± 13.4	0.611
BMI kg/m^2^	28.7 ± 4.5	29.0 ± 5.0	28.1 ± 3.5	28.9 ± 3.9	29.9 ± 4.5	27.6 ± 2.8	0.670
SMM kg	29.0 ± 7.1	25.2 ± 3.2	35.7 ± 7.3	28.9 ± 6.2	24.4 ± 1.0	34.8 ± 5.2	0.802
FAT kg	28.4 ± 9.7	30.5 ± 10.1	24.6 ± 8.1	28.8 ± 9.4	32.5 ± 10.1	23.7 ± 5.5	0.887
TBW l	38.6 ± 8.9	34.0 ± 4.0	47.0 ± 9.1	38.5 ± 7.8	33.0 ± 1.3	45.8 ± 6.7	0.929
LBM kg	52.6 ± 12.1	46.2 ± 5.5	63.9 ± 12.4	52.4 ± 10.4	45.1 ± 1.7	62.3 ± 8.9	0.941
PBF %	34.8 ± 8.1	38.9 ± 6.2	27.5 ± 5.5	35.2 ± 9.2	41.0 ± 7.8	27.5 ± 3.2	0.920
WHR	0.9 ± 0.05	0.9 ± 0.05	0.9 ± 0.05	1.0 ± 0.05	0.9 ± 0.06	1.0 ± 0.03	0.178
VFA cm^2^	13.3 ± 4.7	14.5 ± 4.6	11.2 ± 4.1	13.7 ± 5.3	15.8 ± 5.5	11.0 ± 3.5	0.771

**Notes:** ^a^ Statistically significant differences in mean values of the investigated parameters between both groups, not controlled for age. **Abbreviations:** m—mean, SD—standard deviation, BMI—Body Mass Index, SMM—skeletal muscle mass, FAT—fat mass, TBW—total body water, LBM—lean body mass, PBF—percent body fat, WHR—waist-hip ratio, VFA—visceral fat area.

**Table 3:** Mean differences in body composition between Groups 1 and 2.

Parameters	Mean Differences between Baseline and Last Training Results (m ± SD)				*p* ^a^
	2x/week		3x/week		
Body mass kg	−1.21 ± 2.76	−1.3%	−1.95 ± 3.15	−2.6%	0.258
SMM kg	1.49 ± 8.55	+5.9%	−0.07 ± 1.19	0.0%	0.720
FAT kg	−1.36 ± 3.37	−3.7%	−1.90 ± 2.98	−8.3%	0.368
TBW l	−0.23 ± 2.09	−0.7%	−0.02 ± 1.46	−0.2%	0.461
LBM kg	−0.32 ± 3.13	−0.7%	−0.05 ± 1.98	−0.2%	0.650
BMI kg/m^2^	−0.22 ± 1.07	−0.6%	−0.54 ± 1.03	−2.1%	0.230
PBF %	−0.57 ± 2.22	−1.4%	−1.97 ± 2.89	−6.2%	0.05
WHR	0.00 ± 0.01	0.0%	−0.02 ± 0.02	−1.6%	0.006
VFA cm^2^	−0.73 ± 2.02	−4.0%	−1.14 ± 1.76	−10.0%	0.371

**Notes:** ^a^ Statistical significance of mean differences in body composition at baseline and after the last training in Groups 1 and 2. **Abbreviations:** m—mean, SD—standard deviation, BMI—Body Mass Index, SMM—skeletal muscle mass, FAT—fat mass, TBW—total body water, LBM—lean body mass, PBF—percent body fat, WHR—waist-hip ratio, VFA—visceral fat area.

**Table 4:** Analysis of the impact of the weekly number of workouts on body mass composition.

Parameters	Estimate ^a^	SE	t	*p*
BMI kg/m^2^	0.28	0.54	0.52	0.605
SMM kg	−0.63	0.57	−1.10	0.273
FAT kg	−0.60	1.18	−0.51	0.610
TBW l	−0.71	0.72	−0.99	0.324
LBM kg	−1.01	0.99	−1.02	0.309
PBF %	−0.78	0.86	−0.91	0.364
WHR	0.00	0.01	0.20	0.839
VFA cm^2^	−0.57	0.63	0.90	0.370

**Notes:**^a^ Controlled for sex, age, and number of training weeks. **Abbreviations:** BMI—Body Mass Index, SMM—skeletal muscle mass, FAT—fat mass, TBW—total body water, LBM—lean body mass, PBF—percent body fat, WHR—waist-hip ratio, VFA—visceral fat area.

## Data Availability

The data analyzed during the study are available from the authors on reasonable request.
